# Primary squamous cell carcinoma of the thyroid treated with concurrent chemoradiation and palliative immunotherapy: a case report

**DOI:** 10.1186/s13256-022-03596-0

**Published:** 2022-10-05

**Authors:** Meng-Lun Hsieh, Brian M. Besch, Jo Elle G. Peterson, Christina Henson

**Affiliations:** 1 Department of Radiation Oncology, University of Oklahoma Health Sciences Center, Oklahoma City, OK 73104 USA; 2 Department of Pathology, University of Oklahoma Health Sciences Center, Oklahoma City, OK 73104 USA

**Keywords:** Primary squamous cell carcinoma, Thyroid, Case report, Immunotherapy, Pembrolizumab

## Abstract

**Background:**

Primary squamous cell carcinoma of the thyroid is a very rare malignancy with aggressive growth and poor prognosis. There is currently no consensus for treatment modality, however, most patients with primary squamous cell carcinoma of the thyroid are treated with surgery and adjuvant chemoradiation.

**Case presentation:**

We report a rare case of primary squamous cell carcinoma of the thyroid in a 68-year-old White male who underwent chemoradiation and palliative immunotherapy after declining surgery. He was treated with intensity-modulated radiation therapy to 70 Gy in 35 fractions, with concurrent carboplatin–paclitaxel and palliative pembrolizumab. Local thyroid disease recurrence occurred at 6 months post-chemoradiation, and the patient died at 16 months post-chemoradiation.

**Conclusions:**

This is the first case report demonstrating the use of pembrolizumab as palliative therapy for primary squamous cell carcinoma of the thyroid. Our study also highlights the importance of chemoradiation in decreasing primary mass size and immunotherapy in preventing metastatic disease progression.

## Background

Primary squamous cell carcinoma (PSCC) of the thyroid is a rare and aggressive neoplasm, accounting for < 1% of all thyroid cancers [1]. Some patients have a history of goiter or thyroid diseases including Hashimoto’s thyroiditis [2]. Thought to have arisen from either undifferentiated follicular cells, metaplastic follicular epithelium, or a thyroglossal duct remnant [3–7], the World Health Organization (WHO) has now classified PSCC as a subtype of anaplastic carcinoma due to similar poor prognosis and molecular alteration [8, 9].

Most patients are diagnosed during the seventh decade of life, with a mean age at presentation of 61 years. However, PSCC of the thyroid can occur at any age [2, 10]. According to the Surveillance, Epidemiology, and End Results Program (SEER) 19-registry database, in the USA between 1973 and 2015, PSCC of thyroid has a female to male ratio of 1.4 to 1 [11]. Patients typically present with a rapidly enlarging neck mass and accompanying cervical lymphadenopathy. Obstructive symptoms such as odynophagia, dysphagia, dysphonia, dyspnea, and neck mass may also be present.

Given the aggressive nature of this disease, an advanced disease state with invasion of adjacent organs is common at the time of diagnosis. This highlights the importance of rapid intervention and the need for additional research and studies to help improve prognosis and prolong survival. Prior to initiating treatment, thyroid PSCC must be distinguished from metastatic squamous cell carcinoma, primary head and neck squamous cell carcinoma from another site extending into the thyroid gland, and the tall cell variant of papillary thyroid carcinoma, as treatment modalities differ drastically with each diagnosis [12]. Diagnostic tools include endoscopy, computed tomography (CT) and positron emission tomography (PET) imaging, and microscopic evaluation of tissue samples with immunohistochemical analysis including resection of specimens, preoperative fine needle aspiration (FNA) cytology, and core needle or excision biopsy [13].

For the pathologist, distinguishing between primary and metastatic squamous cell carcinomas of the head and neck can be particularly challenging as the microscopic appearance and immunohistochemical profiles are largely similar from one site of origin to the next [12]. One notable exception in the head and neck region is human papilloma virus (HPV)-related squamous cell carcinoma of the oropharynx, which can be identified by positive staining with p16, a surrogate marker of oropharyngeal HPV infection [14]. Several studies of thyroid PSCC have shown that thyroglobulin and thyroid transcription factor-1 (TTF-1), stains known to be positive in tumors of thyroid origin, are rarely positive in PSCC. However, paired box protein 8 (PAX-8), another marker of thyroid origin, is frequently positive [2], although polyclonal antibodies can detect nonspecific expression while monoclonal antibodies are more specific [15]. Nevertheless, positive PAX-8 staining in a squamous cell carcinoma involving the thyroid strongly supports a primary thyroid origin in much the same way p16 stains positive in HPV-related oropharyngeal squamous cell carcinomas. Interestingly, partial p16 positivity has also been described for PSCC [9].

Due to the rarity of this neoplasm, no consensus on treatment exists. Treatment options include surgery, chemotherapy, and chemoradiation [16]. In this study, we report a case of PSCC of the thyroid in a 68-year-old male with no known prior thyroid disease or head and neck skin cancer who was treated with chemoradiation and palliative immunotherapy. Not only did the patient’s primary tumor decrease in size, demonstrating radiosensitivity and/or chemosensitivity to carboplatin–paclitaxel, but improvements in pulmonary metastases were also seen with pembrolizumab. To our knowledge, this is the first case report to demonstrate the use of pembrolizumab as palliative therapy to PSCC of the thyroid, and highlights the importance of considering chemoradiation with or without immunotherapy as initial first-line treatment over surgery despite a dismal prognosis.

## Case presentation

**Fig. 1:**
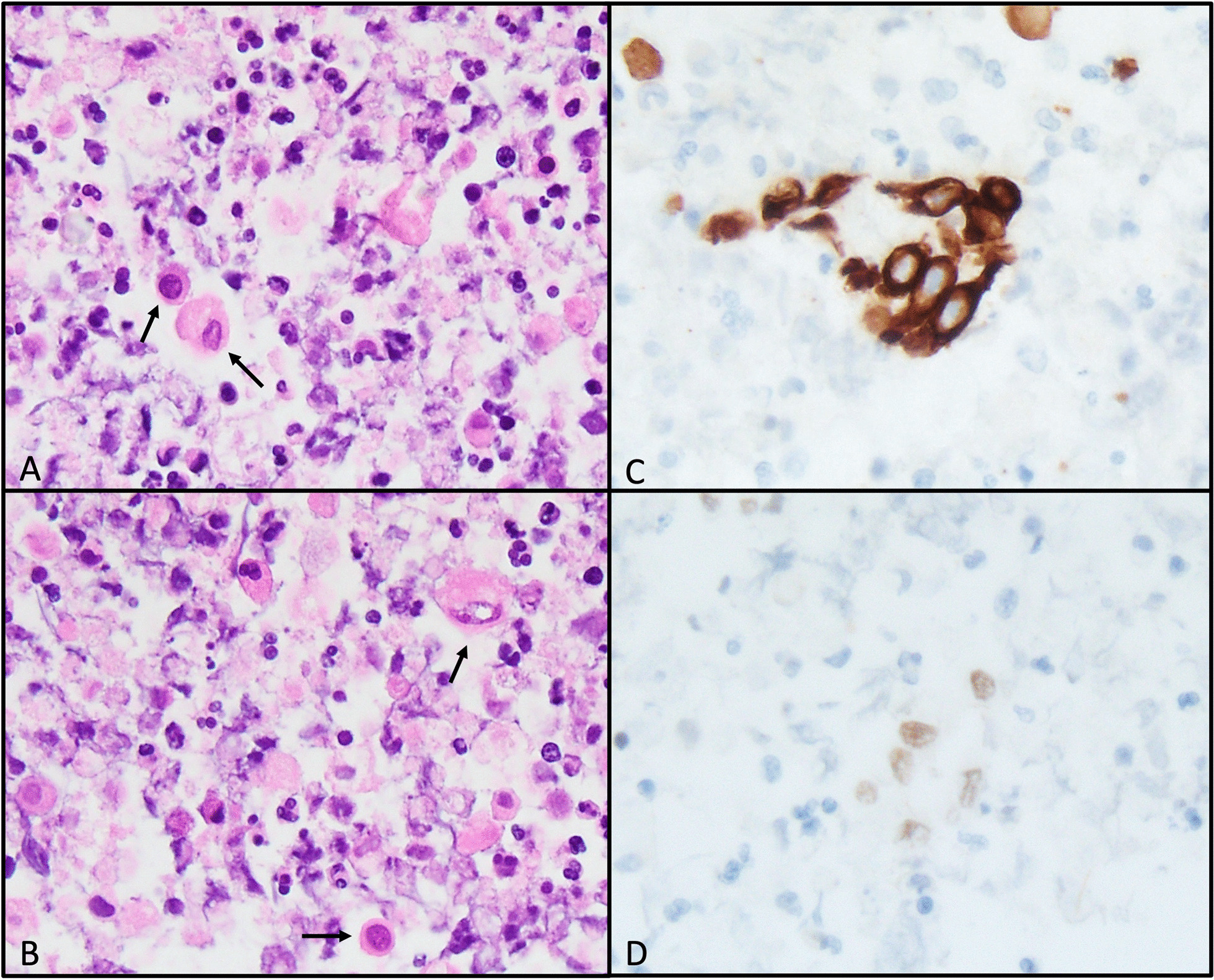
H&E stained cell block slides (**A** and** B**, 60X) demonstrating dysmorphic cells (arrows). CK5/6 immunohistochemical stain (**C**, 60×) with cytoplasmic staining highlighting cells of squamous differentiation. PAX-8 (Biocare Medical, BC12) immunohistochemical stain (**D,** 60×) with nuclear staining of the same cell cluster seen in **C**

**Fig. 2:**
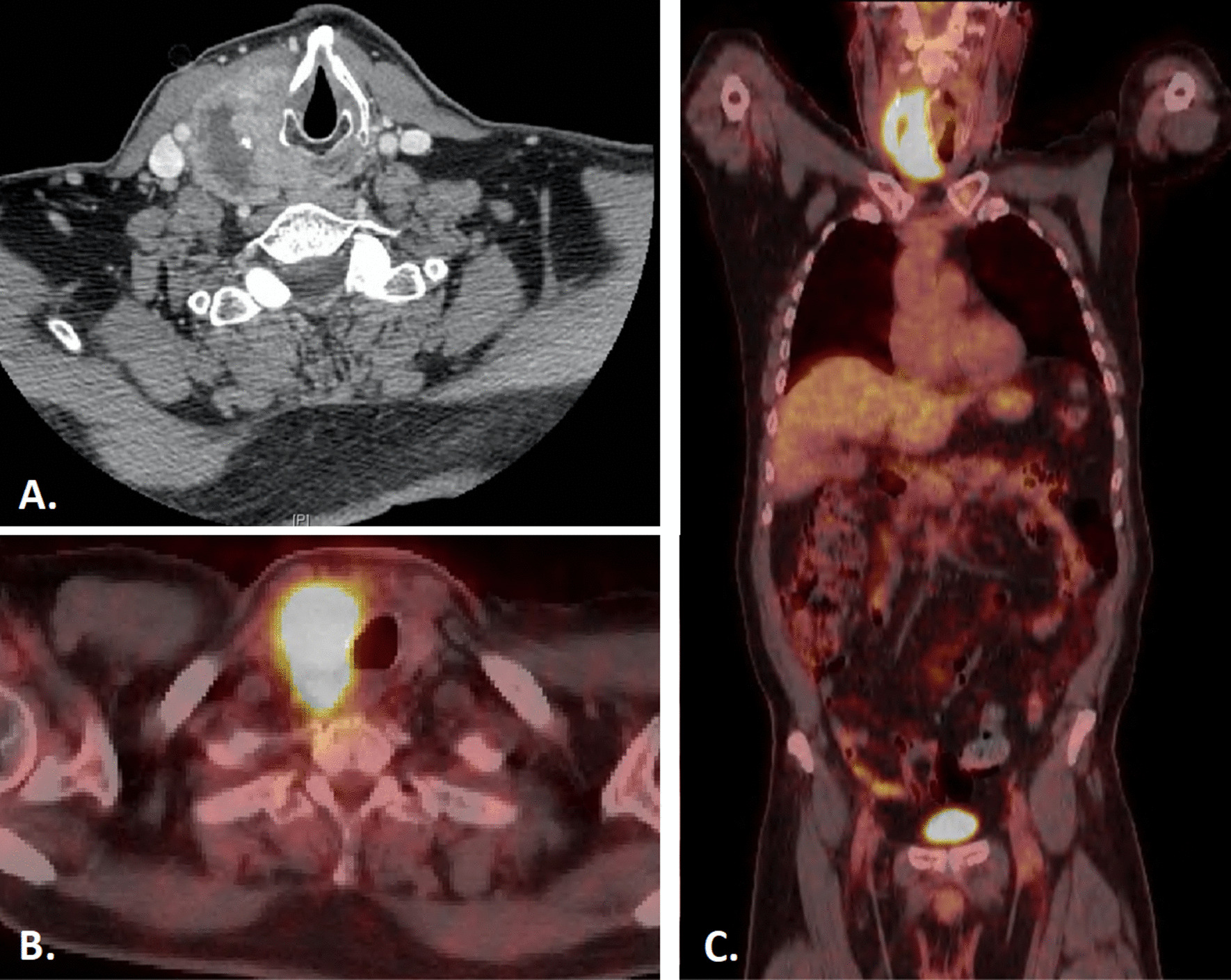
Pre-treatment diagnostic CT neck (**A**) and staging PET/CT (**B**, **C**) scan revealed a large right necrotic and PET-avid thyroid mass

We present a rare and interesting case of a 68-year-old White male with T4aN1bM0, stage III (AJCC 8th edition) squamous cell carcinoma of the thyroid. His past medical history included hypertension, prostate cancer treated with androgen deprivation therapy, external beam radiation therapy, and brachytherapy, and a benign parotid tumor status post-parotidectomy 20 years prior. He was retired from working in corporate management, and family history was significant for lung cancer in his father and breast cancer in a sister. Comorbid illnesses included gastrointestinal reflux disease, for which he took ranitidine 150 mg daily and omeprazole 20 mg daily; hypertension, for which he took lisinopril 12.5 mg daily; hyperlipidemia, for which he took atorvastatin 20 mg daily; and seasonal allergies and asthma, for which he took montelukast 150 mg daily and loratadine 10 mg daily. He was also using naproxen and cannabis for cancer-related pain at time of presentation. He was a lifelong non-smoker and did not drink alcohol to excess. He first noticed his right neck mass while shaving 3 months prior to presentation. He reported some mild pain with swallowing, weight loss of unknown amount, right neck sensitivity to light touch, increased hoarseness, and a raspy voice. He was initially diagnosed by his primary care physician as having lymphadenopathy secondary to a sinus infection and received two rounds of antibiotics. The mass failed to improve and, as a result, a fine needle aspiration of the lesion was performed. Evaluation of the biopsy material demonstrated dysmorphic cells containing cytoplasmic keratin, which stained positively for CK5/6, consistent with a squamous cell carcinoma. Monoclonal PAX-8 (Biocare Medical, BC12) staining was also focally positive (Fig. 1). Subsequent laryngoscopy revealed no mucosal lesions, but right vocal cord paralysis was observed. A CT of the neck with contrast revealed a 4.8 cm peripherally enhancing hypodense mass with central necrosis lateral to the right hyoid bone, emanating from the thyroid gland (Fig. 2A). Multiple smaller nodular lesions within the prevertebral space, right paraesophageal region, and superior mediastinum were also identified. A staging PET scan showed a hypermetabolic right thyroid lobe with extensive conglomerate pathologic adenopathy at the right cervical lymph node chain, extending inferior to the right thoracic inlet, as well as a left retropharyngeal hypermetabolic lymph node, but no evidence of distant metastasis (Fig. 2B and C). On initial physical and neurologic examination, the oncology team noted a large, firm, fixed right medial neck mass without overlying skin changes, and a normal cranial nerve exam.

The patient’s case was discussed at the head and neck multidisciplinary tumor board, with a consensus opinion that this squamous cell carcinoma was indeed of thyroid origin. Extensive surgical resection including a laryngectomy, thyroidectomy, and reconstruction followed by adjuvant chemoradiation as a potential curative course was recommended. However, due to the morbidity and lifestyle implications associated with surgery, the patient declined surgery and instead chose to pursue concurrent chemoradiation with weekly chemotherapy.

**Fig. 3:**
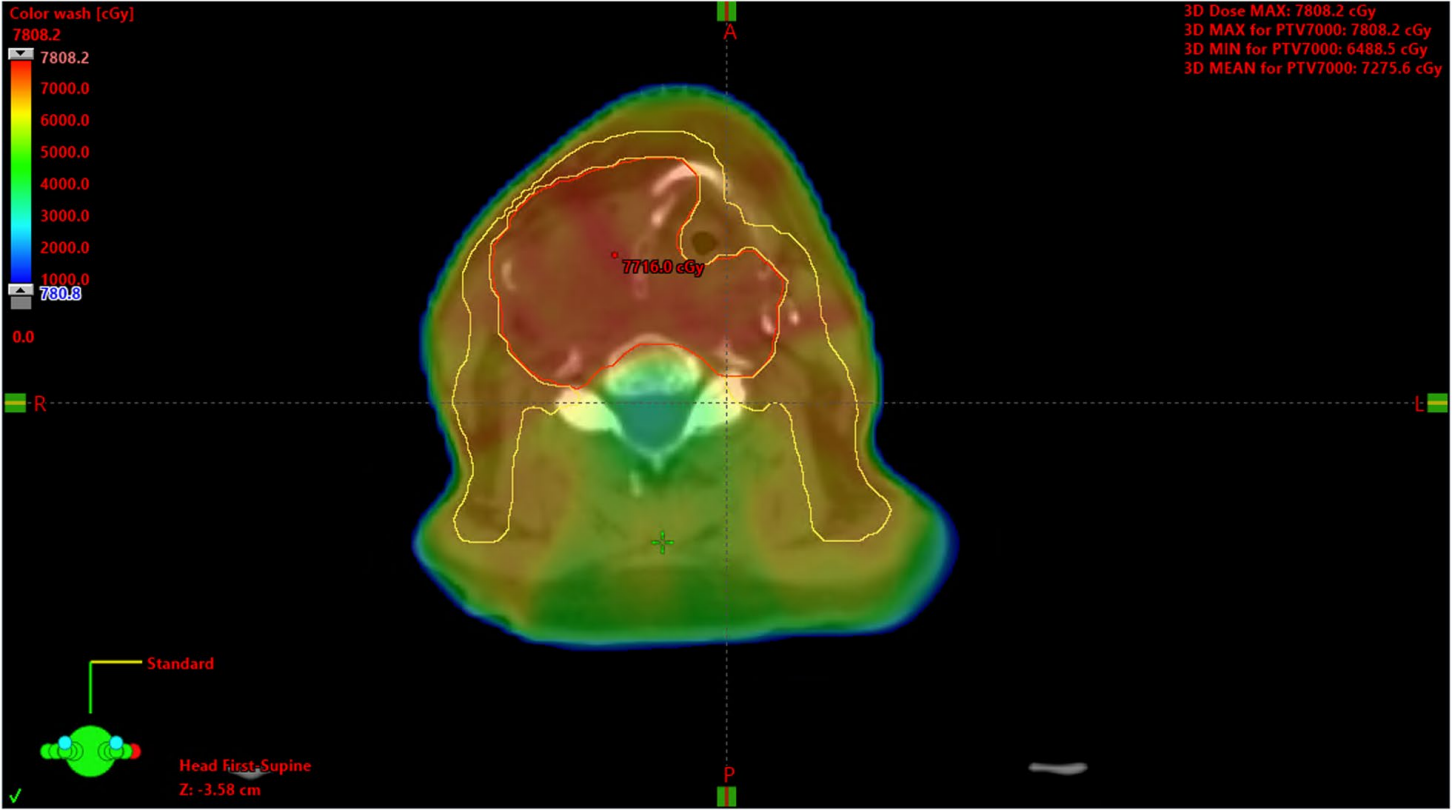
Axial view of patient’s CT-based radiation treatment plan. 6  MV photon beams were utilized, and patient was prescribed to 7000 cGy in 35 fractions to the isocenter. Dose color wash is represented as a percentage relative to the prescribed 7000 cGy dose. Thyroid mass contours are delineated in red and are treated to 7000 cGy, while retropharyngeal and cervical neck nodal levels IB, II, III, and IV are delineated in yellow and treated to 6300 cGy

Intensity modulated radiation therapy (IMRT) was used to treat the right thyroid mass to 70 Gy in 35 fractions at 200 cGy per fraction, as well as bilateral retropharyngeal nodes cervical neck nodal levels IB, II, III, IV, and V to 63 Gy in 35 fractions at 180 cGy per fraction (Fig. 3). The patient completed radiation with weekly concurrent carboplatin-paclitaxel over 7 weeks. During treatment, he developed grade 2 fatigue, grade 2 mucositis, grade 2 dermatitis, grade 3 dysphagia, and grade 3 dry mouth with thickened secretions with excessive mucus. The skin desquamation was treated with silver sulfadiazine and mineral oil-hydrophil petrolat. A percutaneous endoscopic gastrostomy (PEG) tube was placed during the first week of treatment due to nutrition and oral intake difficulties at baseline, and the patient lost less than 15 lbs throughout the treatment. Long-term toxicity was significant for chronic xerostomia and dysphagia, requiring a permanent gastrostomy tube. He also developed treatment-related hypothyroidism and was started on levothyroxine approximately 3 months after completion of treatment, when his thyroid-stimulating hormone (TSH) was found to have gone up from 1.63 (baseline, normal) to 21 mIU/mL (elevated).

**Fig. 4:**
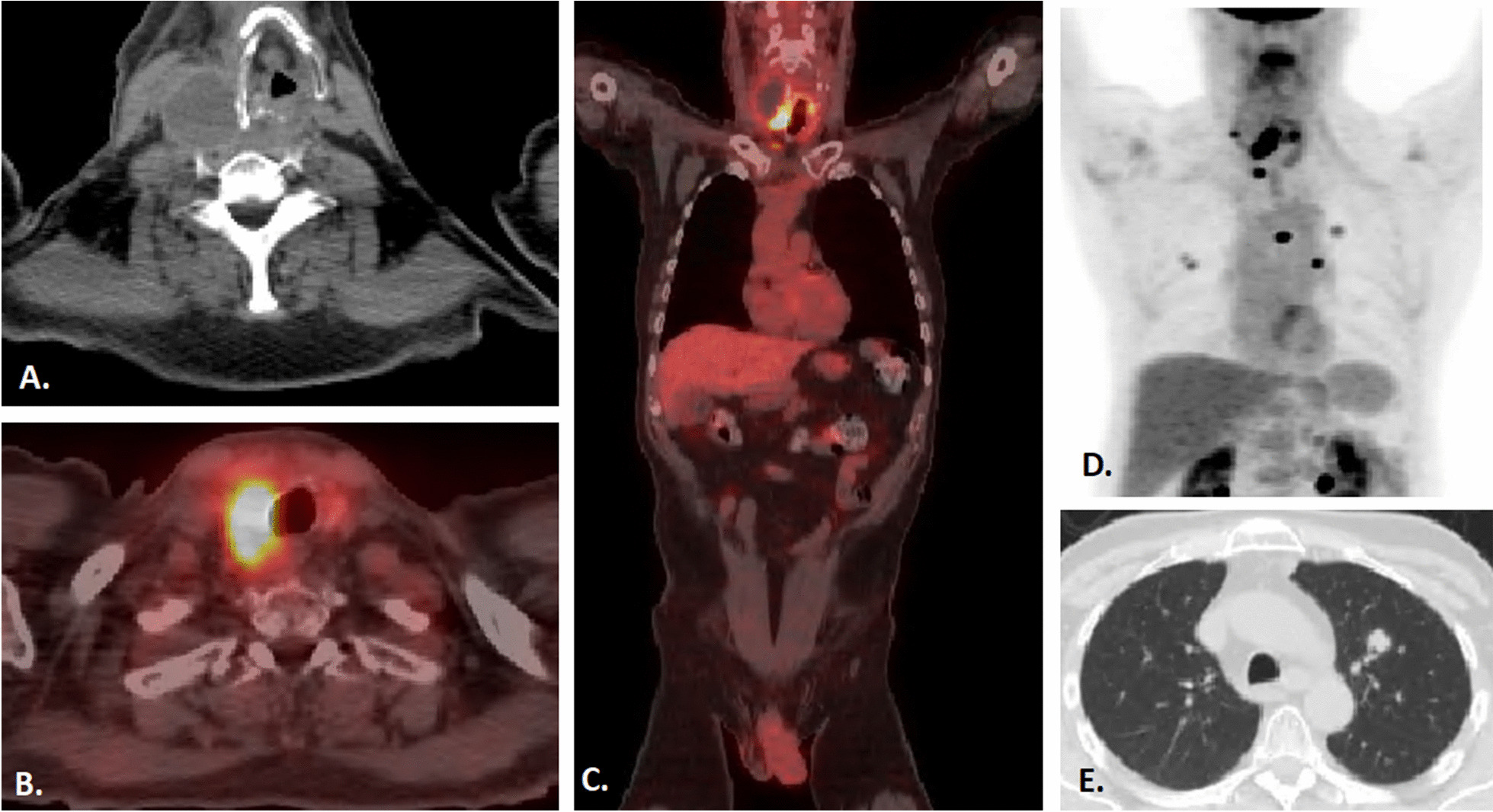
Post-concurrent chemoradiation PET/CT imaging with carboplatin/paclitaxel reveal a smaller thyroid tumor size (**A**) and decreased PET-avidity (**B**, **C**), but show new bilateral pulmonary metastases (**D**, **E**)

After receiving concurrent chemoradiation, the patient underwent two additional cycles of high-dose carboplatin–paclitaxel every 3 weeks. Follow-up imaging after concurrent chemoradiation suggested a partial therapeutic response. PET (18-fluorodeoxyglucose/18-FDG)/CT of the neck demonstrated that the thyroid mass had decreased in size from 5.0 × 5.2 cm, with maximum standardized uptake values (SUV) of 35.3, to 3.8 × 2.1 cm, with maximum SUV of 19.4 (Fig. 4A–C). This was also apparent on physical examination, where the tumor was smaller, softer, and less tender to palpation. The right and left neck lymph nodes were also notably smaller and demonstrated less PET-avidity. Imaging of the chest, however, revealed pulmonary metastases up to 9 mm in size, as well as multiple bilateral hypermetabolic nodules (Fig. 4D and E).

**Fig. 5:**
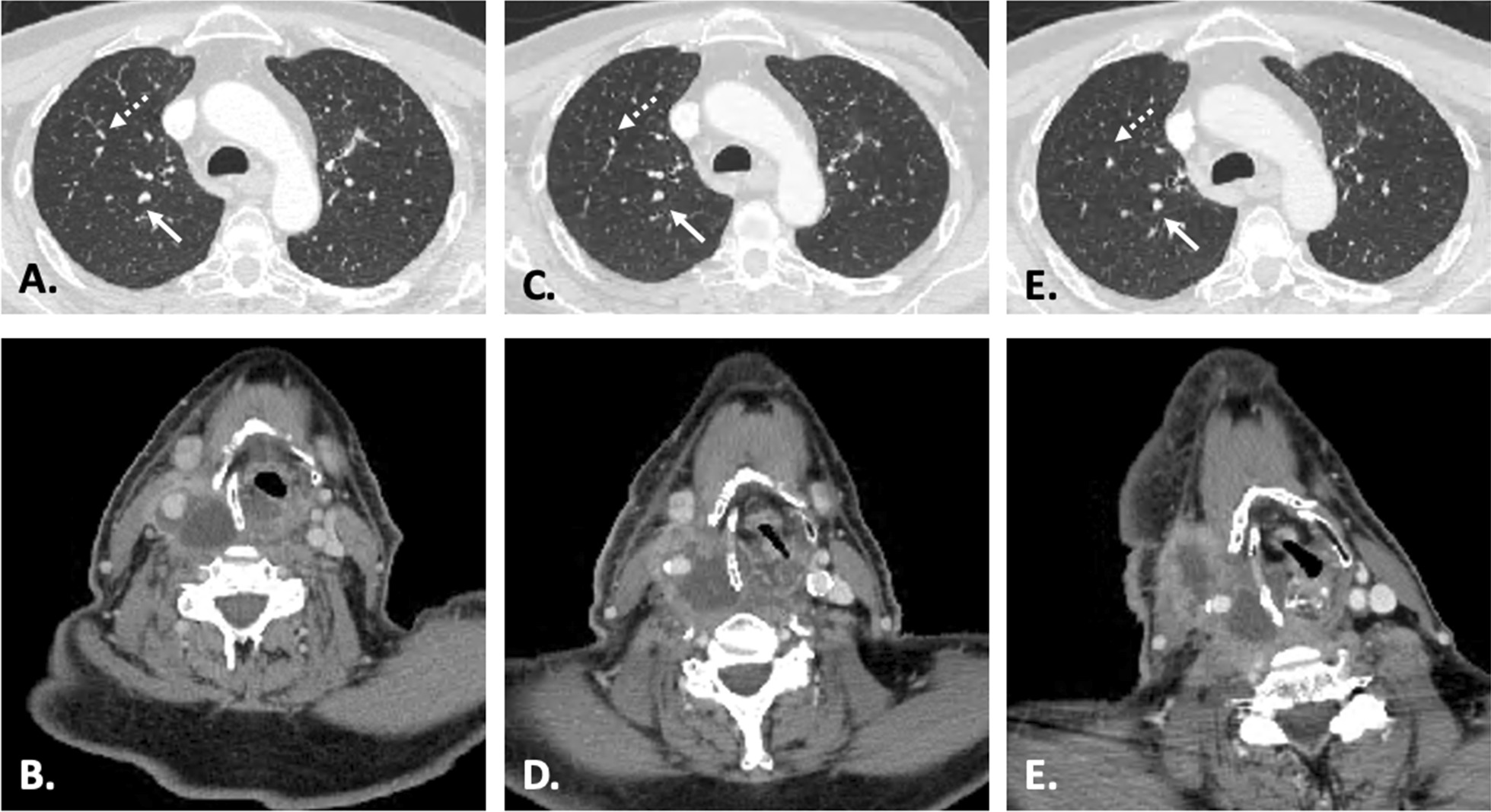
Post-immunotherapy CT imaging demonstrates enlarging thyroid tumor but stable (white arrows) or decreased pulmonary metastases (dotted arrows) after three cycles (**A**, **B**), six cycles (**C**, **D**), and nine cycles (**D**, **E**) of pembrolizumab

In light of the new pulmonary metastatic disease, additional systemic chemotherapy with carboplatin–paclitaxel was recommended to the patient. However, he opted for immunotherapy instead due to previous chemotherapy side effects and subsequently completed nine cycles of pembrolizumab. Imaging studies obtained after the third cycle of pembrolizumab demonstrated an interval decrease in the size of multiple pulmonary nodules bilaterally, suggesting a positive response to therapy (Fig. 5A). At the same time, however, a CT scan demonstrated an increase in the size of the thyroid mass (Fig. 5B). Additionally, new encasement of the distal common carotid artery, carotid bifurcation, and proximal internal carotid artery was present, and erosion of the thyroid cartilage was also identified. After the sixth cycle of pembrolizumab, imaging continued to demonstrate enlargement of the thyroid mass with increased extension to the tracheoesophageal groove, while the pulmonary nodules continued to get smaller (Fig. 5C and D). Following the ninth cycle of pembrolizumab, the thyroid mass had continued to enlarge, eroding the thyroid and cricoid cartilages, extending into the paraglottic space, and invading the cervical esophagus. No new or worsening pulmonary metastatic disease was identified at this time (Fig. 5E and F). The tenth cycle of pembrolizumab was halted due to leukocytosis (WBC 21.81 and ANC 19.05) and the patient’s desire to pursue supportive care. Fifteen months after his initial diagnosis, the patient was transitioned to comfort care and died 3 months later. Autopsy was not performed as the patient’s desire was to donate his body to science.

## Discussion

PSCC of the thyroid is a rare and aggressive cancer with very poor prognosis. Diagnosis and management remain challenging as PSCC of the thyroid represents < 1% of all primary thyroid cancers.

In this case report, we describe a 68-year-old male with PSCC of the thyroid who, after declining surgery, underwent IMRT with concurrent carboplatin–paclitaxel and palliative pembrolizumab. PSCC has been previously shown to be relatively insensitive to chemotherapy and radiation [17–19]. Our case report shows, however, that not only is PSCC responsive to radiation and carboplatin–paclitaxel as demonstrated by both physical examination and PET/CT imaging, but our patient’s pulmonary metastatic disease was responsive to immunotherapy. The size of the thyroid mass was notably decreased on both imaging and physical examination at 3 months post-chemoradiation. At the same time, however, the patient was identified as having pulmonary metastases, at which point immunotherapy was started. While the tumor size progressively increased and demonstrated worsening local invasion starting at 6 months post-chemoradiation, the pulmonary nodules progressively decreased in size during treatment with pembrolizumab.

To our knowledge, this is the first reported use of pembrolizumab in the treatment of PSCC of the thyroid. Pembrolizumab is a highly selective monoclonal IgG4 antibody directed against the programmed cell death protein-1 (PD-1) receptor on the cell surface. For head and neck squamous cell carcinoma, pembrolizumab is second-line treatment after platinum-based chemotherapy. Though we did not see a decrease in the size of the thyroid mass with pembrolizumab, we did observe a significant decrease in the size of pulmonary metastases and did not identify any new pulmonary masses. In addition to pembrolizumab, other targeted immunotherapies with receptor tyrosine kinase inhibitors, such as Lenvatinib, are currently being studied owing to its ability to target multiple angiogenic and carcinogenic signaling pathways [20]. More research is needed to analyze the possible use of concurrent immunotherapy with chemotherapy or concurrent immunotherapy with chemoradiation as treatment options.

A previous systemic literature review of 50 patients with PSCC of the thyroid revealed a dominating radiation prescription of 60 Gy in 30 fractions and platinum-containing chemotherapy. Median survival was 6 months (range 0–40 months) and 81% of patients died within 12 months [18]. Our patient demonstrated local disease recurrence at 6 months post-chemoradiation and died 16 months after chemoradiation and immunotherapy. We believe that treatment of 70 Gy in 35 fractions, weekly carboplatin-paclitaxel, and pembrolizumab played an important role in prolonging the survival of this patient, especially in the setting of no surgery. The review by Struller *et al.* described seven patients with reported disease free survival at 12, 15, 18, 20, 21, 24, and 26 months, respectively; however, it is important to note that these patients had disease limited to the thyroid gland without lymph node involvement and underwent surgical resection followed by postoperative adjuvant therapy with radiation, chemotherapy, or chemoradiation [18].

Molecular alterations have also recently been identified, which led to changes in the WHO classification of PSCC of the thyroid from its own separate entity to a subtype of anaplastic thyroid carcinoma in 2022. In a recent multi-institutional study of over 300 patients from two tertiary centers, PSCC not only had a similar outcome to anaplastic thyroid carcinoma but also displayed the BRAFV600E mutations in 87.5% of cases, regardless of thyroid differentiation status [8, 9]. Our patient was diagnosed in August 2019 and passed away in February 2021 and, unfortunately, no molecular analysis was performed.

There is currently a lack of treatment consensus for PSCC of the thyroid. The most optimal treatment modality appears to be complete surgical resection with adjuvant chemoradiation [17–19, 21], though long-term disease-free survival has yet to be reported. Additional research is needed to evaluate the natural course of the disease with surgery and adjuvant chemoradiation versus chemoradiation alone with or without immunotherapy. Due to the aggressive nature of this malignancy, as well as the high morbidity associated with surgery, we recommend reconsidering chemoradiation with immunotherapy as a treatment addition to definitive standard of care options, despite overall extremely poor prognosis.

## Conclusion

PSCC of the thyroid is a rare and aggressive malignancy with very poor prognosis. With no treatment consensus due to the rarity of the disease, most patients are treated with surgery and adjuvant chemoradiation, despite previous studies demonstrating relative resistance to both chemotherapy and radiation. Our case report highlights the importance of chemoradiation and immunotherapy in PSCC of the thyroid. Not only did the patient’s thyroid mass decrease in size on both physical examination and imaging studies with carboplatin-paclitaxel and 70 Gy of radiation, but his pulmonary metastases also decreased in size and/or remained stable with pembrolizumab. We recommend reconsidering chemoradiation and immunotherapy as a definitive standard of care option when surgery is not an option. Further studies are needed to evaluate the role of immunotherapy in PSCC of the thyroid.

## Data Availability

All of the data and materials will be available upon request to the corresponding author.

## Abbreviations

CT: Computed tomography
CK: Cytoplasmic keratin
FDG: Fluorodeoxyglucose
HPV: Human papilloma virus
IMRT: Intensity-modulated radiation therapy
PAX-8: Paired box protein 8
PEG: Percutaneous endoscopic gastrostomy
PET: Positron emission tomography
PSCC: Primary squamous cell carcinoma
SEER: Surveillance, Epidemiology, and End Results Program
SUV: Standardized uptake values
TTF-1: Thyroid transcription factor-1

## References

[1] Syed MI, Stewart M, Syed S, Dahill S, Adams C, McLellan DR, *et al*. Squamous cell carcinoma of the thyroid gland: primary or secondary disease? J Laryngol Otol. 2011;125(1):3–9. 10.1017/S0022215110002070 20950510

[2] Lam AK. Squamous cell carcinoma of thyroid: a unique type of cancer in World Health Organization classification. Endocr Relat Cancer. 2020;27(6):R177–92. 10.1530/ERC-20-0045 32252028

[3] Goldman RL. Primary squamous cell carcinoma of the thyroid gland: report of a case and review of the literature. Am Surg. 1964;30:247–52. 14149689

[4] Heitz P, Moser H, Staub JJ. Thyroid cancer: a study of 573 thyroid tumors and 161 autopsy cases observed over a thirty-year period. Cancer. 1976;37(5):2329–37. 10.1002/1097-0142(197605)37:5<2329::aid-cncr2820370523>3.0.co;2-7 1260718

[5] Lam KY, Lo CY, Liu MC. Primary squamous cell carcinoma of the thyroid gland: an entity with aggressive clinical behaviour and distinctive cytokeratin expression profiles. Histopathology. 2001;39(3):279–86. 10.1046/j.1365-2559.2001.01207.x 11532039

[6] Sahoo M, Bal CS, Bhatnagar D. Primary squamous-cell carcinoma of the thyroid gland: new evidence in support of follicular epithelial cell origin. Diagn Cytopathol. 2002;27(4):227–31. 10.1002/dc.10178 12357501

[7] Simpson WJ, Carruthers J. Squamous cell carcinoma of the thyroid gland. Am J Surg. 1988;156(1):44–6. 10.1016/s0002-9610(88)80169-7 3394892

[8] Baloch ZW, Asa SL, Barletta JA, Ghossein RA, Juhlin CC, Jung CK, *et al*. Overview of the 2022 WHO classification of thyroid neoplasms. Endocr Pathol. 2022;33(1):27–63. 10.1007/s12022-022-09707-3 35288841

[9] Iwamoto Y, Anno T, Koyama K, Ota Y, Nakashima K, Monobe Y, *et al*. Primary squamous cell carcinoma of the thyroid with severe tracheal invasion: a case report. Eur Thyroid J. 2021;10(6):548–9. 10.1159/000511709 PMC8647117 34950602

[10] Ab Hadi I, Bliss RD, Lennard TW, Welch AR. Primary squamous cell carcinoma of the thyroid gland: a case report and role of radiotherapy. Surgeon. 2007;5(4):249–51. 10.1016/s1479-666x(07)80010-2 17849961

[11] Yang S, Li C, Shi X, Ma B, Xu W, Jiang H, *et al*. Primary squamous cell carcinoma in the thyroid gland: a population-based analysis using the SEER database. World J Surg. 2019;43(5):1249–55. 10.1007/s00268-019-04906-2 30719559

[12] Kleinhans H, Schmid KW, Verse T. Primary squamous cell carcinoma of the thyroid gland. HNO. 2013;61(7):661–3. 10.1007/s00106-012-2622-y 23241865

[13] Tunio MA, Alasiri M, Riaz K, Alshakweer W. Pancreas as delayed site of metastasis from papillary thyroid carcinoma. Case Rep Gastrointest Med. 2013;2013: 386263. 10.1155/2013/386263 PMC3623391 23607002

[14] Robinson M, Sloan P, Shaw R. Refining the diagnosis of oropharyngeal squamous cell carcinoma using human papillomavirus testing. Oral Oncol. 2010;46(7):492–6. 10.1016/j.oraloncology.2010.02.013 20227331

[15] Lai WA, Hang JF, Liu CY, Bai Y, Liu Z, Gu H, *et al*. PAX8 expression in anaplastic thyroid carcinoma is less than those reported in early studies: a multi-institutional study of 182 cases using the monoclonal antibody MRQ-50. Virchows Arch. 2020;476(3):431–7. 10.1007/s00428-019-02708-4 31732814

[16] Shrestha M, Sridhara SK, Leo LJ, Coppit GL 3rd, Ehrhardt NM. Primary squamous cell carcinoma of the thyroid gland: a case report and review. Head Neck. 2013;35(10):E299-303. 10.1002/hed.23152 23002023

[17] Cho JK, Woo SH, Park J, Kim MJ, Jeong HS. Primary squamous cell carcinomas in the thyroid gland: an individual participant data meta-analysis. Cancer Med. 2014;3(5):1396–403. 10.1002/cam4.287 PMC4302690 24995699

[18] Struller F, Senne M, Falch C, Kirschniak A, Konigsrainer A, Muller S. Primary squamous cell carcinoma of the thyroid: case report and systematic review of the literature. Int J Surg Case Rep. 2017;37:36–40. 10.1016/j.ijscr.2017.06.011 PMC5479948 28633125

[19] Wang W, Ouyang Q, Meng C, Jing L, Li X. Treatment optimization and prognostic considerations for primary squamous cell carcinoma of the thyroid. Gland Surg. 2019;8(6):683–90. 10.21037/gs.2019.11.07 PMC6989903 32042676

[20] Yasumatsu R, Sato M, Uchi R, Nakano T, Hashimoto K, Kogo R, *et al*. The treatment and outcome analysis of primary squamous cell carcinoma of the thyroid. Auris Nasus Larynx. 2018;45(3):553–7. 10.1016/j.anl.2017.07.009 28739190

[21] Chen KH, Chou YH, Cheng AL. Primary squamous cell carcinoma of the thyroid with cardiac metastases and right ventricle outflow tract obstruction. J Clin Oncol. 2012;30(26):e260–3. 10.1200/JCO.2011.39.9808 22649151

